# Applying Recurrent Neural Networks for Anomaly Detection in Electrocardiogram Sensor Data

**DOI:** 10.3390/s23249878

**Published:** 2023-12-17

**Authors:** Ana Minic, Luka Jovanovic, Nebojsa Bacanin, Catalin Stoean, Miodrag Zivkovic, Petar Spalevic, Aleksandar Petrovic, Milos Dobrojevic, Ruxandra Stoean

**Affiliations:** 1 Teacher Education Faculty, University of Pristina in Kosovska Mitrovica, 38220 Kosovska Mitrovica, Serbia; ana.minic@pr.ac.rs; 2 Faculty of Informatics and Computing, Singidunum University, 160622 Belgrade, Serbia; luka.jovanovic.191@singimail.rs (L.J.); nbacanin@singidunum.ac.rs (N.B.); mzivkovic@singidunum.ac.rs (M.Z.); aleksandar.petrovic@singidunum.ac.rs (A.P.); mdobrojevic@singidunum.ac.rs (M.D.); 3 Department of Computer Science, Faculty of Sciences, University of Craiova, 200585 Craiova, Romania; rstoean@inf.ucv.ro; 4 Faculty of Technical Science, University of Pristina in Kosovska Mitrovica, Filipa Visnjica bb, 38220 Kosovska Mitrovica, Serbia; petar.spalevic@pr.ac.rs

**Keywords:** electrocardiogram, diagnosis, particle swarm optimization, optimization, recurrent neural networks

## Abstract

Monitoring heart electrical activity is an effective way of detecting existing and developing conditions. This is usually performed as a non-invasive test using a network of up to 12 sensors (electrodes) on the chest and limbs to create an electrocardiogram (ECG). By visually observing these readings, experienced professionals can make accurate diagnoses and, if needed, request further testing. However, the training and experience needed to make accurate diagnoses are significant. This work explores the potential of recurrent neural networks for anomaly detection in ECG readings. Furthermore, to attain the best possible performance for these networks, training parameters, and network architectures are optimized using a modified version of the well-established particle swarm optimization algorithm. The performance of the optimized models is compared to models created by other contemporary optimizers, and the results show significant potential for real-world applications. Further analyses are carried out on the best-performing models to determine feature importance.

## 1. Introduction

Cardiovascular health is a global concern as the leading cause of death globally [1]. Many factors contribute to this, among which is the more static lifestyle that has become a standard for most people, and this is especially troublesome as the lack of physical activity leads to various health issues. Other factors include age, stress, and diet irregularities. People who smoke and drink alcohol regularly are also found to be more prone to heart-related diseases [2]. All these factors can be attributed to personal choices and, as Keeney et al. [3] suggest, around 25% of cardiovascular diseases can be avoided just by altering personal choices without even considering genetics and other factors that influence an individual’s health. Long periods of time are usually required for health conditions regarding cardiovascular health to develop. This is due to their nature, as cardiovascular problems develop over time and increase in severity. Furthermore, such illnesses are harder to spot, which jeopardizes the health of the patient.

Traditional ways of monitoring cardiovascular health are open to improvements, with the emphasis being on the time it takes to diagnose a disease. The heart emits electrical signals that can be monitored by instruments, and the most applied principle is the electrocardiogram (ECG) [4]. The standard system consists of 10 sensors [5] distributed over the human body for a precise reading, but other variations with more sensors exist as well. The primary point for the placement of the sensors is the chest due to the position of the heart. The rest are distributed over the limbs. For example, the standard system with 10 electrodes produces 12 leads, which are represented by waveforms. The activity of the heart from specific angles is represented by leads [6]. The ECG provides a graph of the heartbeat and its rhythm, allowing medical personnel to detect possible irregularities that can be indicators of diseases. The results of the ECG are visual and readable by professionals only, but this is not the only hindrance, as the data can have imperfections due to the nonlinearity and complexity of signals, as well as the low amplitude of recordings, and noise [7].

Considering the previously mentioned shortcomings of ECG systems, possible solutions are being explored to improve the determination of cardiovascular health. As artificial intelligence (AI) techniques are beginning to improve daily aspects of humans’ lives, the medical field is not an exception in this trend [8]. Improvements in ECG systems aim for faster recognition of patterns, leading to quicker diagnoses. This is of great importance as it allows patients to begin treatment sooner and reduces the risk of improper medication, which can occur with manual result interpretation. The ECG data problem is formulated as a time-series task, which makes it suitable for AI applications. As Wolpert et al. [9] state in the “no free lunch theorem” (NFL), not one model is perfect and provides equally optimal results for all problems. For this particular type of problem neural networks provide the best results, as they are suited for time-series problems due to their architecture being modeled after the human nervous system. Regardless of their previously achieved exceptional performance in this field, such solutions are not without shortcomings. This is usually solved by applying an optimization technique that can tune the performance of the main solution by providing the optimal subset of its configuration parameters. Every problem requires customized frameworks as the NFL states, and the combinations of such solutions are vast.

For this study, a recurrent neural network (RNN) is selected as the predictor of heart conditions due to its high performance with time-series prediction tasks [10]. RNNs can form feedforward along with feedback connections, with the latter being activated with a delay to ensure the forming of long-term dependencies. This aspect of the RNN architecture makes them well-suited for detecting heart-related diseases, given that these conditions develop slowly over time. The idea is to provide a model that can allow for early detection of these conditions, which has not been achieved in this manner to date. In this context, early detection and rapid diagnostics are crucial as they allow timely intervention and management, potentially preventing complications and improving patient outcomes. The particle swarm optimization (PSO) algorithm, belonging to the swarm intelligence algorithm family, has been selected as an optimizer for the RNN hyperparameters. By applying this principle, the experimentation can result in a model that is as close as possible to optimal, as defined by the NFL. The problem of health predictions based on waveforms belongs to the group of problems of non-deterministic polynomial time complexity (NP-hard). For this type of problem, swarm metaheuristics have proven excellent optimizers. The proposed method was tested against other RNN models optimized by other high-performing metaheuristics for the purpose of results comparison.

An extensive literature survey has shown that there is a research gap in this domain, particularly, RNN has not been applied with PSO in this domain. Therefore, the primary goal of this manuscript is to apply PSO to tune the RNN’s hyperparameters for this specific problem, aiming to develop a lightweight RNN architecture that is capable of achieving good results in ECG analysis.

The summarized main contributions of this work are provided:

- The proposal of a lightweight solution for an essential issue of cardiovascular health diagnosis through a robust AI-based framework.
- RNN predictor application for the time-series problem of hearth electrical signal waveforms.
- Swarm intelligence PSO algorithm optimizer for the specific point in question of RNN hyperparameter tuning.
- An extensive analysis of high-end metaheuristic optimizers for RNN optimization.

The organization of the sections is briefly provided: Section 2 provides the fundamentals of the research, Section 3 explains the inner workings of the original optimization algorithm and the performed improvements, and Section 4 provides a basis for experimentation. The experimentation outcomes are presented in Section 5. Finally, Section 6 provides a summary of the problem formulation, the accomplishments of the research, and grounds for future work.

## 2. Background

The utilization of AI in the field of medicine has gained significant attention from researchers, primarily driven by various compelling factors. Among these factors, the continually growing demand for healthcare services and the increasing need for rigorous scrutiny during the diagnostic process serve as powerful motivators for researchers to explore the integration of automation into the medical domain [11,12]. Moreover, the evolving landscape of networking and the internet of things (IoT) [13] has generated a heightened demand for enhanced security measures. Applications of IoT networks in combination with AI have shown admirable outcomes when applied to issues associated with healthcare [14,15].

One intriguing area where AI finds practical application is in the realm of time-series analysis. These algorithms enable the observation and prediction of trends within continuous datasets, facilitating the determination of data patterns, directions, and correlations. Algorithms that can effectively account for temporal aspects within data have exhibited promising results when applied to complex real-world challenges [16,17]. Furthermore, advanced data decomposition techniques have been combined with time-series data, further enhancing their performance by breaking down signals into a series of component signals. This approach often leads to improved forecasting outcomes, as complex signals are inherently challenging to predict, while a series of simpler signals can be more readily managed and analyzed [18,19].

### 2.1. AI Approaches in Electrocardiogram Analysis

AI methods have been increasingly employed in the analysis and interpretation of ECGs to aid in the diagnosis of cardiovascular diseases [20,21]. CNNs are effective for image-based tasks, and ECG signals can be treated as 1D images. CNNs can automatically learn hierarchical features from ECG data and can be useful in routine clinical practice, as shown by [22,23,24].

RNNs, and their variant long short-term memory networks (LSTMs), are useful for capturing temporal dependencies in ECG signals, making them suitable for tasks such as arrhythmia detection. These models are typically lightweight, simple, and show promising efficiency and accuracy, as discussed by [25,26]. Hybrid methods have also been considered, such as the CNN-LSTM approach introduced in [27].

Machine learning algorithms have been considered for this problem as well [28,29]. Random forests and decision trees may be used for classification tasks, such as identifying different types of arrhythmia [30,31,32]. On the other hand, support vector machines (SVMs) may be effective for binary classification tasks and have been applied to identify specific cardiac conditions in ECGs [33].

### 2.2. Recurrent Neural Networks

With the goal of creating a neural network that is more suitable for problems that require sequential data analysis, the RNN was created. The difference from the basic neural network is the existence of recurrent connections between neurons, allowing for future inputs memory storage. Sequential layers with neurons are connected similarly to the basic neural network, as well as the weights and biases for connection input evaluation, decision-making, and output generation. RNNs require optimization in terms of architecture in addition to the control parameters for optimal performance. The benefits of using RNNs less complex in their structure are observable in data interpretation and training. More complex architecture is required for problems that have to keep track of complex nonlinear relationships.

The hidden state in the previous time step $h_{t-1}$ is combined with the current at the time step *t*. The process is described by Equation (1).

(1) $$a_{t}=b+Wh_{t-1}+Ux_{t},$$

where $a_{t}$ is the input activation, *b* the bias term, and *W* and *U* represent the weight matrices of recurrent and input connections, respectively.

Equation (2) describes the process of the alteration of the hidden state after every input with the ϕ activation function over the $a_{t}$.

(2) $$h_{t}=\phi \left(a_{t}\right)$$

Based on the prediction goal, different functions can be used as ϕ. The output of the network is derived from the hidden state. The previously described process is mathematically formulated by Equation (3).

(3) $$y_{t}=Wh_{t}$$

While RNNs have a unique ability of working with and reacting to changes in sequential data, certain drawbacks can be observed in the basic model. This class of networks is particularly sensitive to vanishing and exploding gradients [34], making good models difficult to construct. Furthermore, RNNs can only retain an influence of one previous input, which can limit their applicability for longer term forecasts. Certain methods have been developed to deal with these issues. such as the gated recurrent unit (GRU) and long short-term memory networks (LSTMs). However, while these versions of RNNs offer some advantages, they come at the cost of an increased complexity relative to the base algorithm.

### 2.3. Metaheuristics

The field of metaheuristic algorithms became popular due to the algorithms’ proficiency in solving NP-hard problems. The main challenge is to find solutions to these problems within a reasonable timeframe, while also maintaining reasonable hardware requirements. The algorithms can be divided further into subgroups, but there is no formal definition. The grouping that is recognized by most researchers includes the differentiation by the phenomena used for inspiration of the algorithm. In this manner, the different groups include swarm, genetic, physics, human, and the most novel group of these, the mathematically inspired algorithms.

Swarm-inspired solutions take inspiration from species that live in large groups and the aspects of their lives that benefit from group behavior [35]. This is often the case when a single unit is incapable of completing a task on its own, and that is where other units of the same species come into play. The swarm group of algorithms has provided excellent results with solutions to NP-hard problems, but to reach their maximum potential, hybridization with similar solutions is advised. The issue of these stochastic population-based algorithms is that they usually favor one of the two phases between exploration and exploitation, which can be overcome by incorporating a mechanism from a different solution. Notable algorithms from the swarm family include PSO [36], genetic algorithm (GA) [37], sine cosine algorithm (SCA) [38], firefly algorithm (FA) [39], grey wolf optimizer (GWO) [40], reptile search algorithm (RSA) [41], as well as the COLSHADE [42] algorithm.

Swarm metaheuristics find application in a wide range of real-world problems. Some of the implementations include glioma MRI classification [43], detection of credit card fraud [44,45], global optimization problems and engineering optimization [46,47,48], cloud computing [49], prediction of the number of COVID-19 cases [50], feature selection [51], and wireless sensor networks [52,53].

Ahmadpour et al. [54] developed a genetic-algorithm-based solution to track subjects’ blood pressure, significantly improving their overall quality of life and allowing for the early detection of preventable diseases. Khan et al. [55] explore an IoT environment that enables all-day monitoring of patients’ conditions and greatly improves their cardiovascular health.

Examples of AI-assisted medical diagnosis include diabetic retinopathy detection [56], skin lesion classification [57], lung cancer classification [58], and magnetic resonance imaging (MRI), among diverse other applications in medicine.

Although it is one of the first metaheuristics algorithms, proposed over twenty years ago, PSO is still considered a very powerful optimizer. Recently, the PSO algorithm has been successfully implemented, either in a basic or modified version, to tackle numerous problems in the medical and other domains. Notable examples include tuning LSTM for ECG-based biometric analysis [59], CNN-based classification of cardiac arrhytmias and healthcare monitoring [60,61], and RNN-based cloud balancing [62], to mention a few.

## 3. Methods

The following section describes the base algorithm that serves as a basis for modification. The algorithm is selected empirically, based on previous research where significant potential has been observed. Following the description of the basic algorithm, its initialization, and search mechanisms, we highlight observed limitations and present a potential solution. Finally, the pseudocode of the final modified approach is provided.

### 3.1. The Original PSO

The original PSO was introduced in 1995 by Kennedy and Eberhart [36]. The flocking of birds and fish was the main inspiration for this metaheuristic. Particles are represented as search agents and are considered a part of the population. Discrete as well as continuous problems can be solved by the PSO.

The model of the algorithm works such that every particle is assigned an initial velocity, which can be regarded as the position in the population. During one iteration the particles change their location in search of a better one. The weight component describes how fast the particles move. The weights are the old velocity, the best obtained so far, and the best yet obtained by the neighboring particle.

(4) $$\left\{\begin{array}{c} \vec{v_{i}}\leftarrow \vec{v_{i}}+\vec{U}\left(0,\phi_{1}\right)⨂\left(\vec{p_{i}}-\vec{x_{i}}\right)+\vec{U}\left(0,\phi_{2}\right)⨂\left(\vec{p_{g}}-\vec{x_{i}}\right)\\ \vec{x_{i}}\leftarrow \vec{x_{i}}+\vec{v_{i}}, \end{array}\right.$$

In Equation (4), the component-wise multiplier is shown as ⨂, the range of all the components from $v_{i}$ is $\left[-V_{max},+V_{max}\right]$, and the vector $\vec{U}\left(0,\phi_{1}\right)$ represents every particle randomly generated and uniformly distributed in the range $\left[0,\phi_{i}\right]$. The value of $p_{i}$ denotes the best solution for particle *i* while pg denotes the global best particle. Any of the particles is a possible solution in a *D*-dimensional space and its position is defined by Equation (5), the best position obtained before the update is shown in Equation (6), and the velocities are given in Equation (7):

(5) $$X_{i}=\left(x_{i1},\;x_{i2},\;\ldots ,\;x_{iD}\right)$$

(6) $$P_{i}=\left(p_{i1},\;p_{i2},\;\ldots ,\;p_{iD}\right)$$

(7) $$V_{i}=\left(v_{i1},\;v_{i2},\;\ldots ,\;v_{iD}\right)$$

The best solutions overall and in the group are noted as $p_{i}$ and $p_{g}$, respectively. The search agent takes both pieces of information into account before deciding on the next move in terms of the distance currently between its position and $p_{i}$ and $p_{g}$.

With the application of the inertia weight approach, this behavior can be modeled as in Equation (8):

(8) $$v_{id}=W*v_{id}+c_{1}*r_{1}*\left(P_{id}-X_{id}\right)+c_{2}*r_{2}*\left(P_{gd}-X_{id}\right)$$

The relative influence inertia factors are shown in Equation (8) as *w*, $c_{1}$, and $c_{2}$, used for cognitive and social components, respectively. $r_{1}$ and $r_{2}$ are random numbers, while the particle velocity and the current position are given, respectively, as $v_{id}$ and $x_{id}$. $p_{id}$ and $p_{gd}$ are the $p_{i}$ and $p_{g}$, respectively.

Equation (9) describes the inertia factor. $w_{max}$ is the initial weight, while $w_{min}$ is the final weight; *T* is the maximum number of iterations, and the current iterations are given as *t*.

(9) $$w=w_{max}-\frac{w_{max}-w_{min}}{T}\cdot t$$

### 3.2. Genetically Inspired PSO (GIPSO)

Although the original PSO shows good performance, it exhibits certain shortcomings when evaluated using standard CEC [63] evaluation functions. To address these issues and enhance the PSO, this study introduces hybridization techniques. Drawing inspiration from the genetic algorithm (GA) [37], we create a new algorithm known as the genetically inspired PSO (GIPSO).

In the GIPSO algorithm, a novel introduction mechanism is activated after each iteration. It selects a random agent and combines it with the best solution obtained so far. The algorithm uniformly combines their parameters, and this combination is governed by a control parameter, denoted as pc. Empirically, the optimal value for pc has been determined to be 0.1. Furthermore, an additional modification involves parameter mutation. When triggered, this process selects a random value within a specified parameter constraint. Half of this selected value is either added to or subtracted from the parameter, depending on the mutation direction parameter md. Once again, the value for md is determined empirically, set to 0.1.

After generating a new solution, the worst-performing solution in the swarm is replaced by the new agent. The evaluation of the new solution is deferred until the next iteration, maintaining the computational complexity of the original algorithm.

From a mathematical perspective, the introduced algorithm follows the randomness influence initialization pattern of the the PSO algorithm as described in the original study [36]. Updates are also also performed as described in the original algorithm. Each agent *A* contains a vector of values that represent the genetic structure as

(10) $$A_{i}=\left(a_{1},a_{2},\ldots a_{D}\right)$$

where $A_{i}$ represents a given agent and *a* a given parameter and *D* the number of parameters dependent on the dimensionality of the search space. Once crossover is initialized, two agents are selected and recombined:

(11) $$c_{j}=\alpha \cdot a_{j}+\left(1-\alpha \right)\cdot b_{j}$$

where $c_{j}$ denotes the resulting child parameter, $a_{j}$ and $b_{j}$ are the *j*-th parameters of agents *A* and a randomly selected second agent *B*, and α is a random factor. The agent parameters are then further mutated as

(12) $$A_{ik}=A_{ik}+md\cdot rnd\;\mathrm{or}\;A_{ik}=A_{ik}-md\cdot rnd$$

This equation represents the mutation of the *k*-th parameter in an agent, where md is the mutation direction parameter, and rnd is a random value within a specified parameter constraint. The mutation can either add or subtract half of rnd from the original parameter value, depending on the mutation direction. Once an agent has been combined and mutated, the worst solution in the population based on an objective function is replaced. The objective function f(obj) can be adjusted depending on the optimization problem being tackled.

To provide a comprehensive understanding of the algorithm, we present its pseudocode in Algorithm 1.
**Algorithm 1** Pseudocode of the introduced GIPSO. Initialize a population, denoted as *P* **while**
*t* is less than *T* **do**  Evaluate the solutions in *P* using the objective function  **for** Each solution *X* in *P* **do**   Update agent locations by applying the PSO search   Generate a new solution, referred to as NS, using a genetically inspired mechanism   Mutate the parameters of NS   Replace the worst solution in *P* with NS  **end for** **end while** **return** The best solution attained within *P*

The computational complexity of the introduced algorithm remains the same relative to the original as evaluations are only carried out after a new solution has been generated and the worst option replaced. Nevertheless, it is important to note that the implementation complexity of the introduced modified version might be slightly higher compared to the original. However, this potential drawback is considered acceptable when considering the increased boosts to performance.

## 4. Experimental Setup

**Figure 1:**
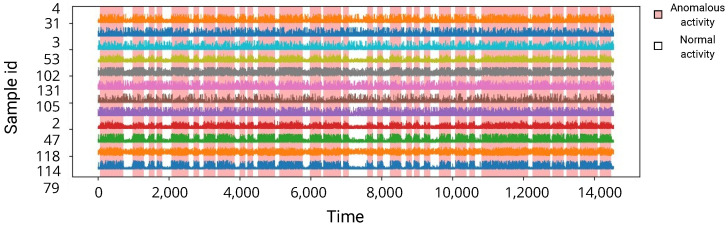
Dataset visualization.

The dataset exploited in this research is the heart rate time series from the Massachusetts Institute of Technology (MIT), publicly available online (https://www.kaggle.com/datasets/ahmadsaeed1007/heart-rate-time-series-mitbih-database, accessed on 13 December 2023). The data are prepared for ML processing and consist of 1800 measurements evenly spaced at intervals of 0.5 s, measured for up to a total of 15 min of monitoring. Readings are captured from 12 sensors (electrodes) on the chest. A visualization of the dataset can be observed in Figure 1. The shown features are time steps that have shown the highest importance following the best-model analysis described later in this work. Normal activity is indicated by a white background while anomalous activity has a red background. Anomalous activity can be considered as any irregular or abnormal heartbeat, otherwise known as an arrhythmia [64].

In the experimentation, 15 lags of data, approximately equivalent to $\frac{1}{8}$ of a second of EEG readings, are used. These inputs are provided to 15 input neurons of an RNN. The number of hidden layers is optimized within [1, 3], favoring lighter networks in order to reduce computational demands, and neuron counts in each of the hidden layers are selected in a range of $\left[\frac{lags}{2},\;lags\right]$. In this research, neuron optimization is, therefore, carried out in a range of [8, 15]. The training parameters are also optimized. The number of training epochs is tuned from the range [30, 60], learning rate from the range [0.01, 0.0001], and dropout from [0.2, 0.05].

The large search space presented by the hyperparameters warrants the use of algorithms capable of effectively addressing complex optimizations. Several contemporary optimization metaheuristics are employed alongside the introduced GIPSO. The metaheuristics are implemented under identical conditions with a population size of 6, and allocated 8 iterations to improve the population quality. To account for variations due to random factors associated with metaheuristic algorithms, the experiments are repeated over 30 independent runs. This also provides a base for further statistical validation of the outcomes. The algorithms included in the comparative performance analysis include the original algorithms used as inspiration, the PSO [36] and GA [37] algorithms, as well as other well-established optimizers, the FA [39], SCA [38] GWO [40], RSA [41], and COLSHADE [42] algorithms.

During the experiments, the initial 70% of the data are used to train the models with parameters selected by the metaheuristic algorithms. A subsequent 10% of the remaining data are used as validation data. The constructed models are repeatedly evaluated using the validation dataset, and their control parameters updated accordingly using metaheuristics. To ensure a valid comparison, the remaining 20% of the data are reserved only for testing. The best constructed models are verified to ensure that no over-fitting has occurred using the elbow method, and early stopping is used to help prevent over-training, with a patience of one-third of the total number of selected epochs.

To provide a comprehensive assessment of the constructed models in comparison to those constructed by other contemporary optimizers, a battery of standard classification metrics including accuracy, precision, recall, and F1 score [65] are utilized. The error rate is used as the objective function for optimization, determined as Error=1−accuracy. Further metrics include Cohen’s kappa [66], described in Equation (13), which gives a more complete assessment in cases when unbalanced datasets are utilized:

(13) $$\kappa =\frac{z_{o}-z_{e}}{1-z_{e}}$$

in which $z_{o}$ and $z_{e}$ represent the observed and expected classification values. Cohen’s kappa is used as the indicator function during the optimizations.

**Figure 2:**
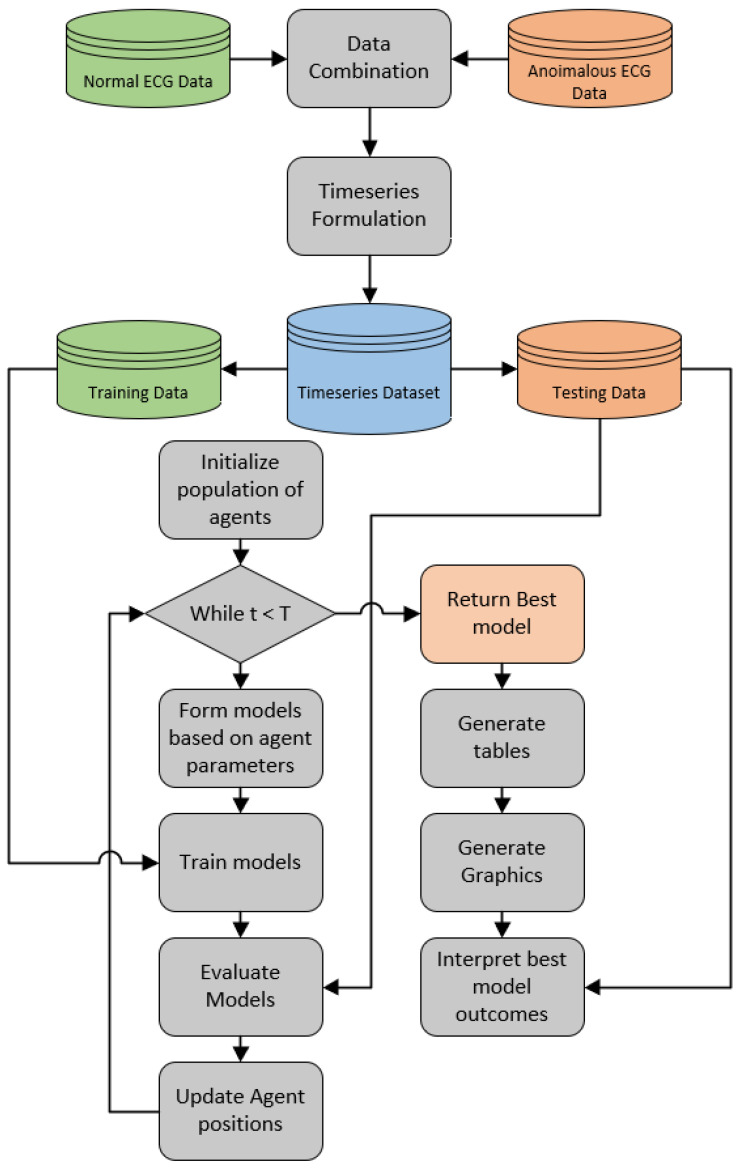
Experimental framework flowchart.

Finally, Figure 2 illustrates the experimental framework flowchart.

## 5. Experimental Outcomes

The experimental outcomes, in terms of the best and worst, as well as the mean and median outcomes throughout 30 independent executions, are provided in Table 1. Further outcomes, in terms of the standard deviation and variance that demonstrate the stability of each optimum, are also provided. Indicator function outcomes are showcased in the same format in Table 2.

Overall the metrics indicate that the introduced GIPSO algorithm attained the best outcome in the best-case scenario. However, the relatively novel RSA shows an impressive performance, attaining better outcomes for the worst-case execution, and thereby showing better outcomes in terms of the mean and median. This improvement carries over to algorithm stability, with the RSA demonstrating higher levels of stability in terms of objective as well as indicator functions. Nevertheless, the modifications applied to the PSO algorithm demonstrate improvements, with the GIPSO algorithm outperforming the original algorithm as well as the GA across all test cases and showing a significant improvement in terms of stability.

**Figure 3:**
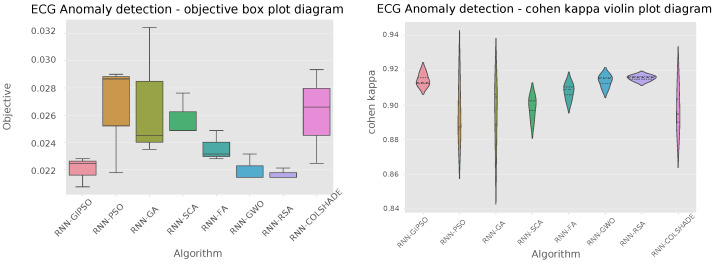
Objective and indicator function distributions.

Comparisons in terms of algorithm stability across the objective and indicator functions are further reinforced by the outcomes shown in Figure 3.

Clear stability improvements over the best PSO and GA can be observed for the GIPSO algorithm. However, the admirable performance of the RSA also needs to be noted, as the metaheuristic demonstrated impressive stability in comparison to competing metaheuristics.

**Figure 4:**
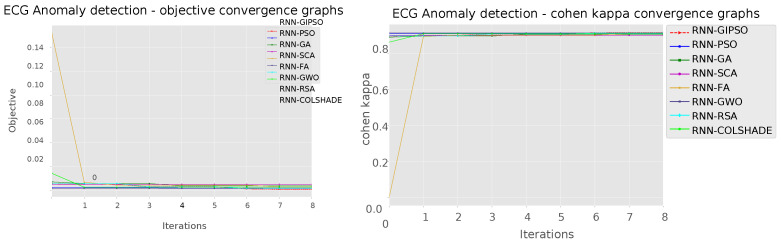
Objective and indicator function convergence graphs.

Improvements in the convergence can be observed in the convergence graphs for the best-performing models shown in Figure 4.

It is important to note that the exploitation power of the algorithm plays a significant factor in this optimization. And it is evident that the introduced algorithm managed to avoid local optima and locate the most promising region within the local search space that presents the best outcomes. Further, detailed metrics for the best-performing models formulated by each metaheuristic are provided in Table 3.

**Figure 5:**
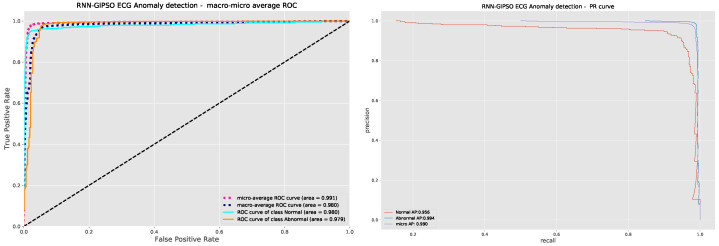
Best-performing model’s ROC and PR curves.

**Figure 6:**
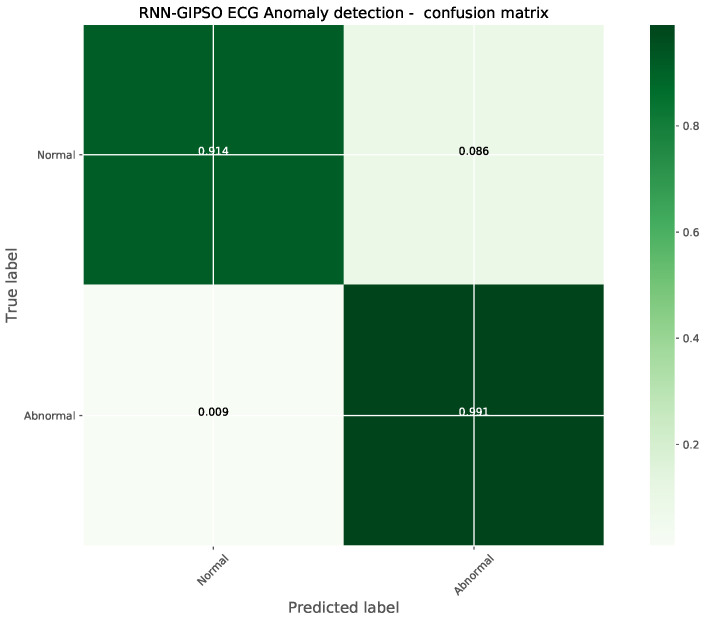
Best-performing model’s confusion matrix.

As can be observed in Table 3, the best-performing model constructed by the introduced metaheuristic demonstrates a clear superiority in comparison to other metaheuristics, attaining the best evaluation score across all but one metric. The best-performing model is further assessed with the ROC and PR curves demonstrated in Figure 5 and the confusion matrix in Figure 6.

It can be deduced that the introduced algorithms constructed a model with parameters optimized for the task of ECG anomaly detection, altering the model to the provided data in such a way that the attained classification accuracy for normal activity reached 91.4% with misclassifications occurring in only 8.6% of cases. Anomalous activity is detected with an even higher accuracy of 99.1%, with misclassifications occurring in only 0.9% of cases.

To encourage experimental repeatability by independent researchers, the parameters selected by each optimization algorithm for their respective best-performing model are provided in Table 4.

### 5.1. Statistical Validation of Outcomes

Because experimental results alone are frequently insufficient to state that one algorithm surpasses its competitors, scientists in modern computer research must establish if the offered enhancements are statistically significant. This study put eight techniques for configuring RNN networks for time-series classification of aberrant cardiac activity to the test, including the proposed GIPSO metaheuristics.

According to the recommendations in [67], statistical tests in such scenarios should involve creating a representative collection of outcomes for each method, which involves creating a sample of outcomes by determining average objective values over several independent executions for each problem. However, this technique may not be appropriate when dealing with outliers that originate from a non-normal distribution, perhaps leading to misleading findings. According to [67], an unresolved debate remains about whether using the mean objective function value in statistical tests is acceptable for comparing stochastic approaches. Despite these possible disadvantages, the classification error rate objective function was averaged across 30 separate runs to compare 10 approaches for detecting ECG anomalies.

After performing the Shapiro–Wilk test [68] for single-problem analysis using the described procedure, a decision was made: a data sample was constructed for each algorithm and each problem by gathering the results of each run, and the corresponding *p*-values were computed for all method–problem combinations. Table 5 shows the resultant *p*-values.

**Figure 7:**
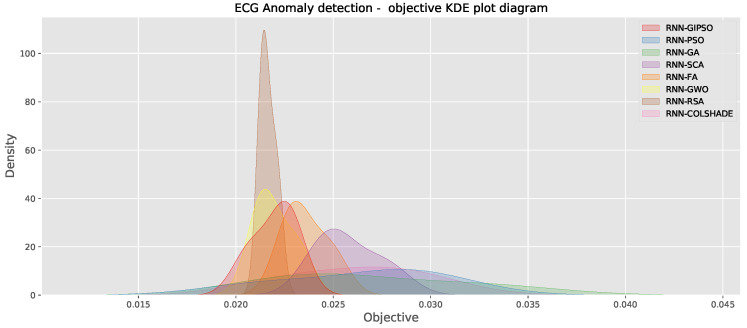
Objective function outcome KDE plots for each metaheuristic.

These outcomes are further enforced in Figure 7, showing the distributions of objective function outcomes of each optimizer over 30 independent runs.

The null hypothesis may be rejected since the *p*-values in Table 5 are all less than the significance threshold, denoted as α, which is set to 0.05. As a result, the data samples for solutions do not all come from the Gaussian distribution, implying that using the average objective value in future statistical tests is not appropriate. As a consequence, the best results were used for further statistical analysis in this study. Because the normalcy assumption was not met, parametric tests were inapplicable. As a consequence, in the next stage, the non-parametric Wilcoxon signed-rank [69] test was utilized. This test can be conducted on the same data series that contains the best values obtained in each metaheuristic run.

In this test, the developed algorithm serves as the control algorithm, and the Wilcoxon signed-rank test was performed on the supplied data series. In all three observed occurrences, the estimated *p*-values were less than 0.05. Using the alpha=0.1 significance threshold, these findings reveal that the new algorithm statistically outperformed all competing techniques by a significant margin. The total results of the Wilcoxon signed-rank test are shown in Table 6.

### 5.2. Best Model Interpretation

It is increasingly important to build trust in ML models, especially when tackling important topics such as healthcare and diagnosis. Further model analysis can highlight issues with the model and help improve methods of data collection. There are several methods for tackling model interpretation. Determining model sensitivity is one approach. However, when dealing with complex multi-layer networks it is often helpful to consider methods that apply model approximations.

**Figure 8:**
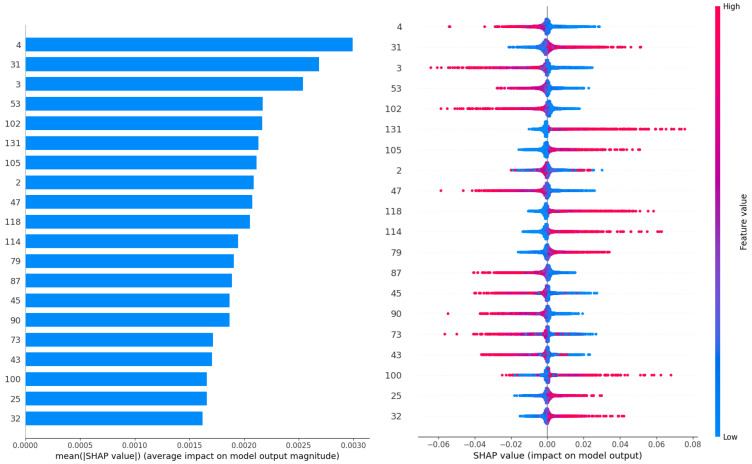
SHAP analysis summary and feature impact outcomes.

The Shapley additive explanations (SHAP) [70] technique relies on game theory in order to build a better understanding of features and their impact on model decisions. Furthermore, this unique approach allows us to consider individual interactions between feature contributions. This work applies the Python implementation of SHAP to analyze the best-constructed anomaly detection model. The outcomes are demonstrated in Figure 8.

The impact of the values in each step of the ECG sequence can be observed in the interpretation outcomes. Each individual sample in the ECG is numerically labeled. It can be observed that early samples have the highest influence of anomalous ECG activity, latter sequences also indicate abnormal readings. However, a fairly consistent importance is observed across all features.

## 6. Conclusions

The conducted research applied metaheuristic algorithms in order to optimize an RNN architecture and training parameters in order to construct models that demonstrate the best results when applied to ECG anomaly detection and classification. A total of seven contemporary metaheuristics were assessed for their ability to optimize hyperparameters and the constructed models were applied and evaluated on a publicly available real-world dataset. An additional modified metaheuristic was introduced that combined ideas borrowed from the GA and incorporated them into the PSO algorithm to improve performance. Several metrics were included in order to provide in-depth comparisons between algorithms. The introduced algorithm created the single best-performing model, outperforming the base PSO algorithm as well as the GA, attaining an accuracy of 91.8695%. Improvements to the exploration and exploitation power of the algorithm were observed in the modified metaheuristic. Additionally, it is important to note the observed great performance of the RSA as well, which demonstrated good performance despite not attaining an optimal model. The constructed models were rigorously statistically validated to confirm the improvements, and the best-performing model was subjected to rigorous sensitivity analysis to attain further insight into the decision-making process.

Some limitations exist within this research. Due to limited computational resources, smaller populations and limited executions were carried out. Only a small subset of available optimization algorithms were evaluated on this problem. Additionally, the capabilities of RNN variants such as LSTM and GRU were explored due to computational constraints. These algorithms present a potential future point of focus in subsequent works. Nevertheless, the proposed method may be used in clinical monitoring for near-real-time analysis. The hardware dictates the execution speed much more than the algorithm itself. Additionally, the execution period is negligibly small compared to optimization. Results may be obtained almost in real-time; only the initial lag must pass as a delay.

In future works wehope to address some of the limitations of this research, expand on the available set of tools for experts and care providers, and offer a better diagnostic technique. Additionally, the potential of recognizing and providing specific disease diagnoses will be explored to further enhance the clinical utility of the model. Furthermore, the potential of the introduced modified algorithm will be explored on other pressing optimization challenges. Finally, modified versions of the RSA will be explored for tackling optimization for ECG data in combination with other algorithms, as significant potential has been observed in this research.

## Figures and Tables

**Table 1:** Objective function outcomes over 30 independent runs. Best results are written in bold.

Method	Best	Worst	Mean	Median	Std	Var
RNN-GIPSO	**0.020718**	0.022790	0.021984	0.022445	0.000906	8.21 × 10${}^{-7}$
RNN-PSO	0.021754	0.029006	0.026473	0.028660	0.003340	1.12 × 10${}^{-5}$
RNN-GA	0.023481	0.032459	0.026819	0.024517	0.004010	1.61 × 10${}^{-5}$
RNN-SCA	0.024862	0.027624	0.025783	0.024862	0.001302	1.70 × 10${}^{-6}$
RNN-FA	0.022790	0.024862	0.023596	0.023135	0.000906	8.21 × 10${}^{-7}$
RNN-GWO	0.021409	0.023135	0.021984	0.021409	0.000814	6.62 × 10${}^{-7}$
RNN-RSA	0.021409	**0.022099**	**0.021639**	0.021409	**0.000326**	**1.06** × **10**${}^{-7}$
RNN-COLSHADE	0.022445	0.029351	0.026128	0.026588	0.002838	8.05 × 10${}^{-6}$

**Table 2:** Indicator function outcomes over 30 independent runs. Best results are written in bold.

Method	Best	Worst	Mean	Median	Std	Var
RNN-GIPSO	**0.918695**	0.912862	0.914520	0.912862	0.002973	8.84 × 10${}^{-6}$
RNN-PSO	0.915662	0.884873	0.895986	0.887422	0.013952	1.95 × 10${}^{-4}$
RNN-GA	0.907855	0.873338	0.895263	0.904598	0.015561	2.42 × 10${}^{-4}$
RNN-SCA	0.902434	0.891184	0.898684	0.902434	0.005303	2.81 × 10${}^{-5}$
RNN-FA	0.911728	0.902800	0.907884	0.909124	0.003749	1.41 × 10${}^{-5}$
RNN-GWO	0.916143	0.909804	0.913765	0.915348	0.002819	7.95 × 10${}^{-6}$
RNN-RSA	0.915985	**0.913923**	**0.915662**	**0.915985**	**0.001308**	**1.71** × **10**${}^{-6}$
RNN-COLSHADE	0.912168	0.885572	0.897502	0.894767	0.011029	1.22 × 10${}^{-4}$

**Table 3:** Detailed metrics of the best-performing models. Best results are written in bold.

Method	Metric	Normal	Anomalous	Accuracy	Macro Avg	Weighted Avg
RNN-GIPSO	Precision	**0.948357**	0.984615	**0.979282**	**0.966486**	**0.979081**
	Recall	0.914027	**0.991035**	**0.979282**	0.952531	**0.979282**
	F1 score	**0.930876**	**0.987815**	**0.979282**	**0.959345**	**0.979124**
RNN-PSO	Precision	0.931663	**0.986569**	0.978246	0.959116	0.978189
	Recall	0.925339	0.987775	0.978246	**0.956557**	0.978246
	F1 score	0.928490	0.987172	0.978246	0.957831	0.978215
RNN-GA	Precision	0.938967	0.982996	0.976519	0.960982	0.976276
	Recall	0.904977	0.989405	0.976519	0.947191	0.976519
	F1 score	0.921659	0.986190	0.976519	0.953925	0.976341
RNN-SCA	Precision	0.800000	0.800000	0.902434	0.890642	0.902434
	Recall	**0.995475**	0.802434	0.902434	0.890368	0.902696
	F1 score	0.887096	0.801214	0.902434	0.890458	0.902860
RNN-FA	Precision	0.927273	0.986156	0.977210	0.956715	0.977169
	Recall	0.923077	0.986960	0.977210	0.955018	0.977210
	F1 score	0.925170	0.986558	0.977210	0.955864	0.977189
RNN-GWO	Precision	0.906143	0.926253	0.916143	0.904172	0.916143
	Recall	0.970588	0.926445	0.916143	0.916517	0.916207
	F1 score	0.904225	0.926348	0.916143	0.911682	0.916567
RNN-RSA	Precision	0.946009	0.984211	0.978591	0.965110	0.978380
	Recall	0.911765	0.990628	0.978591	0.951196	0.978591
	F1 score	0.928571	0.987409	0.978591	0.957990	0.978429
RNN-COLSHADE	Precision	0.939394	0.984191	0.977555	0.961793	0.977354
	Recall	0.911765	0.989405	0.977555	0.950585	0.977555
	F1 score	0.925373	0.986791	0.977555	0.956082	0.977417
	Support	442	2454			

**Table 4:** Indicator function outcomes over 30 independent runs.

Method	Learning Rate	Dropout	Epochs	Layers	Neurons L1	Neurons L2
RNN-GIPSO	0.007225	0.150573	58	1	12	/
RNN-PSO	0.008380	0.102960	60	2	15	12
RNN-GA	0.010000	0.050000	54	1	15	/
RNN-SCA	0.007023	0.185083	53	1	8	/
RNN-FA	0.010000	0.200000	49	2	15	15
RNN-GWO	0.003352	0.198888	53	1	13	/
RNN-RSA	0.007580	0.078741	53	2	14	15
RNN-COLSHADE	0.008961	0.112279	48	1	5	/

**Table 5:** Shapiro–Wilk test scores for the single-problem analysis.

Problem	GIPSO	PSO	GA	SCA	FA	GWO	RSA	COLSHADE
ECG	0.024	0.026	0.017	0.021	0.029	0.035	0.031	0.039

**Table 6:** Wilcoxon signed-rank test values exhibiting *p*-values for experiments (GIPSO vs. others).

Problem	PSO	GA	SCA	FA	GWO	RSA	COLSHADE
ECG-RNN	0.003	0.018	0.037	0.04	0.027	0.031	0.028

## Data Availability

The dataset utilized in this research is the heart rate time series from the Massachusetts Institute of Technology (MIT), which is publicly available online (https://www.kaggle.com/datasets/ahmadsaeed1007/heart-rate-time-series-mitbih-database, accessed on 13 December 2023). All data supporting the reported results can be accessed via the provided link.

## Abbreviations

The following abbreviations are used in this manuscript:

RNN	Recurrent neural network
ECG	Electrocardiogram
GA	Genetic algorithm
PSO	Particle swarm optimization
RSA	Random search algorithm
LSTM	Long short-term memory
GRU	Gated recurrent unit
SCA	Simulated annealing
FA	Firefly algorithm
GWO	Grey wolf optimization
RSA	Random search algorithm
